# Toroidal coil chromatography: The effect of scale-up and “g” field on stage efficiency

**DOI:** 10.1016/j.chroma.2010.12.090

**Published:** 2011-09-09

**Authors:** Ian Sutherland, Peter Hewitson, Joost de Folter

**Affiliations:** Brunel Institute for Bioengineering, Brunel University, Kingston Lane, Uxbridge, Middlesex UB8 3PH, UK

**Keywords:** Process scale, Toroidal coil chromatography, TCC

## Abstract

Selected test results have been taken from various publications and resolution and stage efficiency measured using an established model. All experiments used the same sample and, where possible, the same sample loading. The results show that stage mixing efficiencies have increased from 1.1% in 1998 to greater than 25% in the latest scaled-up version of a Toroidal coil chromatography (TCC) instrument working at 240 g.

## 1 Introduction

Toroidal coil chromatography (TCC) is proving to be a very effective way of purifying proteins using aqueous two-phase systems (ATPS). As it is a hydrostatic counter-current chromatography (CCC) process with a defined number of stages, it lends itself to analysis using a counter-current distribution (CCD) based modelling system [1,2]. The challenge in the future will be to extend the range of CCC instruments to work on the isolation and production of biologics or larger molecules. These are not very stable in organic solvents and require gentle phase systems with a low interfacial tension such as aqueous two-phase systems (ATPS). Ito and Ma [3] demonstrated a separation of lysozyme and myoglobin in a toroidal coil centrifuge (TCC) as early as 1998, but the quantities were small and throughputs very low. Shinomiya et al. [4] later showed that higher resolution separations were feasible using the cross-axis coil planet centrifuge with similar tubing and flow rates as Ito, although he wound his tubing onto a 5 mm core rather than the 3 mm core used by Ito. Later in our own BBSRC funded study (BB/C506364/1) we were able to show that hydrodynamic CCC with wave mixing could give excellent retentions of stationary phase in the column, but when model separation studies were performed it was found that there was poor resolution [5]. It was concluded that wave mixing was too gentle when working with these considerably more viscous solutions. The BBSRC study then went on to show that centrifugal partition chromatography (CPC), another form of liquid–liquid chromatography, which uses a series of chambers mounted circumferentially on a disk interconnected with input and outlet leads and cascade mixing between the phases, could successfully separate the proteins and could be scaled up to larger CPC centrifuges [6]. However the study also showed that there was a large carry-over of the stationary phase (i.e., the process was not an equilibrium one). Also, the process was not completely sterile, as there were rotating seals and the interconnecting chambers were difficult to completely clean between runs. This carry-over was not found when toroidal coils were used instead of spirally wound coils on a multilayer coil planet centrifuge [7,8]. This paper compares the stage efficiencies from 5 different papers using 5 different hydrostatic^1^ CCC columns – a centrifugal TCC column [3]; a cross-axis coil planet centrifuge [4], two multilayer coil planet centrifuge TCC columns [7,8] and a centrifugal partition chromatography (CPC) column [6] – and specifically looks at the effects of increasing “g” field in the TCC columns.

## 2 Modelling

In all cases, chromatograms have been fitted using the theoretical model based on CCD theory [1,2,9], with the equivalent number of mixing and settling transfers (as if the experiment were being performed using a series of liquid–liquid extraction steps in test tubes). Ideally, each mixing stage (or loop with a cascade mixing step or stage) should be 100% efficient, with complete mass transfer between each stage. In practice, the mobile phase often tracks round the path of least resistance and misses the large volumes of stationary phase present. In hydrostatic CCC, where there is cascade mixing like a series of waterfalls, there is an effect known as the “Coriolis Effect” [3] which causes the mixing stream to curve in the direction of rotation when the heavy phase is mobile; this curve varies according to the strength of the “g” field. From the theoretical model [9], knowing the partition coefficients of the sample mixture, myoglobin and lysozyme (*K*_d_ = 0.6 and 1.9), and knowing the retention (*S*_f_), it is possible to find the number of transfers (or mixing and settling steps) to achieve the given resolution (*R*_s_). Efficiency is then obtained by dividing this number by the fixed number of loops/chambers or mixing and settling stages in the instrument being evaluated.

## 3 Preparative scale toroidal coil bobbin

**Fig. 1:**
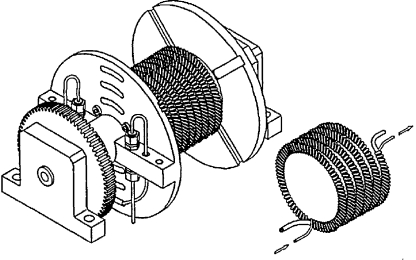
Schematic of toroidal coil bobbin used by Shinomiya et al. [4]. The Brunel toroidal coil uses a similar configuration but wound on a commercial bobbin supplied by Dynamic Extractions with a 334 ml coil volume; 5 mm bore and 404 loops.

A pair of preparative scale stainless steel toroidal coil columns (334 ml coil volume; 5 mm bore and 404 loops each column [8]) are installed in a commercial high performance Midi rotor (supplied by Dynamic Extractions, Slough, UK). These columns and rotor can therefore be operated at up to 240 g. The winding configuration is similar to that used by Shinomiya et al. [4] for his cross-axis rotor (Fig. 1). As the stainless steel tubing was thin walled, the overall weight increase in each completed bobbin was only 6.7% when compared to a standard high performance Midi-bobbin (8.0 kg cf. 7.5 kg), so the mechanical robustness of the overall centrifuge system was not compromised by the use of stainless steel instead of fluoropolymer tubing.

The use of stainless steel for the coils means back pressure generated by the hydrostatic coils can be accommodated. The maximum pressure recorded for the coils in operation was 21 bar (∼300 psi), a backpressure that is within the working pressure of the PTFE flying leads on the Midi instrument, which connect the bobbin to the peripheral equipment.

## 4 Experimental

### 4.1 Sample preparation

The sample was prepared by mixing 2.2 mg/ml each of lysozyme and myoglobin in equal quantities of upper and lower phase (exceptions 10 mg/ml for [3] and 1 mg/ml for [7]).

### 4.2 Sample loading

Sample loading was from 1 to 1.5% of the coil volume (exceptions 5% for [3,4] and 10% for [6]).

### 4.3 Phase system

The phase systems in all cases were 12.5% (w/w) PEG-1000:12.5% (w/w) K_2_HPO_4_ ATPS with the heavy phase mobile.

## 5 Results and discussion

The results in Table 1 show that the mixing efficiency in Ito's conventional toroidal coil centrifuge with its uniform acceleration field and small bore tubing is very low. Shinomiya in using a cross-axis coil planet centrifuge with a non-uniform acceleration field managed to increase the stage efficiency from 1.1 to 7.9% in the same bore tubing. In our PTFE 1.6 mm bore tubing wound on Midi bobbins, where there is also a fluctuating acceleration field, the efficiency is between 2.5 and 3.7% depending on the flow. As the bore increases to 5 mm, with the same rotor speed and increasing the flow from 1.25 to 10 ml/min in the same ratio as the cross section areas of the tubing (linear scale up), the efficiency of mixing increases from 2.5 to 7.4%. This occurred despite the sample loading increasing from 1 mg/ml at 1% coil volume sample loading to 2.2 mg/ml at 1.5% coil volume sample loading. When scaling up to larger bore tubing, the helix angle of the toroid inevitably increases. This means that even higher speeds will be needed to bend the flow stream (Coriolis) into the centre of the tubing and maximise mass transfer from the cascade mixing stream. When rotation speed is increased, there is a dramatic increase in stage efficiency from 7.4% at 800 rpm to 25% at 1400 rpm.

**Fig. 2:**
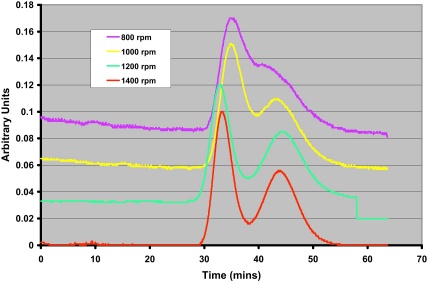
Visualisation of the change in separation resolution with rotational speed changing from 800 rpm (76 g) to 1400 rpm (240 g) [8].

**Fig. 3:**
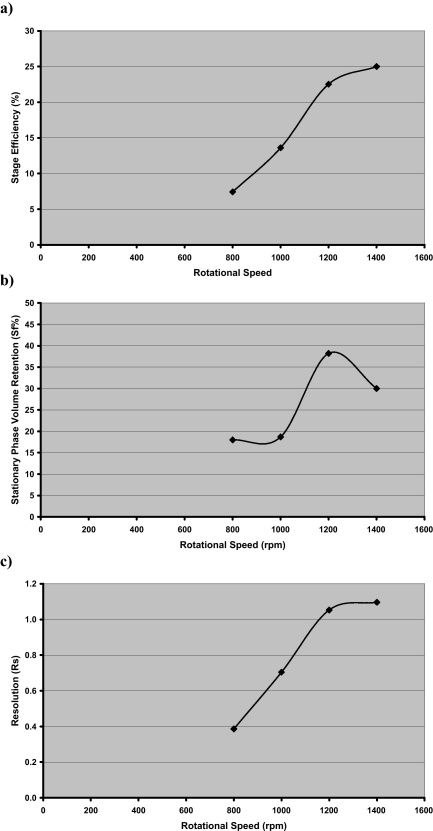
Variation of (a) stage efficiency; (b) stationary phase volume retention (*S*_f_) and (c) resolution (*R*_s_) with rotational speed.

This is can be seen clearly in Fig. 2 where the chromatograms for 800, 1000, 1200 and 1400 rpm have been superimposed upon one another. While the number of theoretical transfers (which is proportional to efficiency) rises significantly with speed (Fig. 3a), the resolution appears to plateau (Fig. 3b). This is because stationary phase retention (*S*_f_) reduces from 38% at 1200 rpm to 30% at 1400 rpm (Fig. 3c).

**Fig. 4:**
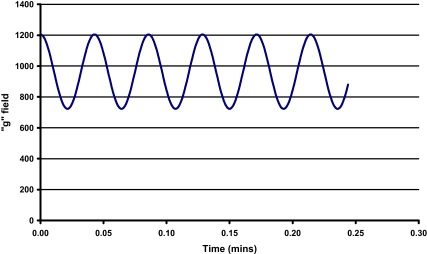
Variation of TCC acceleration field (*x* unit gravity) with time at a constant rotational speed of 1400 rpm.

Ito and Ma [3], when observing “Coriolis flow” in toroidal coils made the following observation: “the best partition efficiency may be obtained when the Coriolis flow is nearly parallel to the toroidal segment where the partition process is taking place”. The huge increase in efficiency observed here is attributed to this effect where at 800 rpm the flow stream is most probably running down the tubing wall and as the speed increases from 800 to 1200 rpm lifts off the wall to become parallel with the wall and at 1400 rpm either starts to hit the opposite wall or as the stream has higher kinetic energy, starts to emulsify – hence the loss of stationary phase. In Ito's case he had a constant acceleration field. While the mean acceleration field at 1400 rpm is 240 g the actual acceleration field in the “J” type coil planet centrifuge fluctuates as shown in Fig. 4 between 720 and 1200 g in the active part of the column. This will result in a huge fluctuation of the cascade mixing stream in both speed and direction facilitating excellent mixing and mass transfer. While the optimum in this case appears to be 1200 rpm there will be a range of operation between 1200 and 1400 rpm where optimum conditions can be achieved.

## 6 Conclusions

Step change increases in stage efficiency have been demonstrated in a preparative toroidal coil stainless steel column with stage efficiencies increasing from 7.4 to 25% and resolution (*R*_s_) from 0.39 to 1.1 as rotational speed increases from 800 rpm (79 g) to 1400 rpm (241 g). These efficiencies are compared very favourably against other technology using the same model system.

## Figures and Tables

**Table 1:** Selection of results comparing the new 5 mm bore TCC instrument with Ito's original TCC unit [3], Shinomiya's cross axis coil planet centrifuge [4], early feasibility studies at Brunel with 1.6 mm bore tubing [7], scale-up with the 5 mm bore tubing [8] and CPC [6].

Centrifuge	Speed (rpm)	Flow (ml/min)	Volume (ml)	Mixing stages	No. of transfers	Mixing efficiency (%)	Resolution (*R*_s_)	Plates (*N*_1_)	Plates (*N*_2_)
TOC [3]	1200	0.2	20	1310	15	1.1	0.96	121	57
XCCC [4]	800	0.2	29	887	70	7.9	1.40		
CCC [7]	800	0.62	51.7	909	28	3.1	0.94	111	39
CCC [7]	800	1.25	51.7	909	23	2.5	0.77	151	37
CCC [7]	800	0.62	107.1	1884	70	3.7	1.45	178	110
CCC [8]	800	10	333.6	404	30	7.4	0.39	340	57
CCC [8]	1000	10	333.6	404	55	13.6	0.70	400	108
CCC [8]	1200	10	333.6	404	91	22.5	1.05	324	145
CCC [8]	1400	10	333.6	404	101	25.0	1.10	380	200
CCC [8]	1400	10	672.1	814	226	27.8	1.68	665	381
CPC [6]	2000	10	429	1008	80	7.9	1.28	308	224

## Footnotes

1 Where “hydrostatic” means that the stationary phase stays where it is when the flow stops, as opposed to “hydrodynamic” where the phases move to opposite ends of the column. Hydrostatic columns have cascade mixing and hydrodynamic columns have wave mixing.
